# Challenges of nursing research with immigrant and refugee populations: methodological and pragmatic considerations

**DOI:** 10.1590/1980-220X-REEUSP-2023-0417en

**Published:** 2024-05-20

**Authors:** Mayckel da Silva Barreto, Gabriel Zanin Sanguino, Viviane Cazetta de Lima Vieira, Mara Cristina Ribeiro Furlan, Gabriel Guembarski Flavio, Andrés Gutiérrez-Carmona, Sonia Silva Marcon

**Affiliations:** 1 Universidade Estadual de Maringá, Departamento de Enfermagem, Maringá, PR, Brazil.; 2 Universidade Federal de Mato Grosso do Sul, Três Lagoas, MS, Brazil.; 3 Universidad de Antofagasta, Departamento de Enfermería, Antofagasta, AN, Chile.

**Keywords:** Emigrants and Immigrants, Refugees, Nursing, Nursing Research, Research Promotion, Emigrantes e Inmigrantes, Refugiados, Enfermería, Investigación en Enfermería, Promoción de la Investigación, Emigrantes e Imigrantes, Refugiados, Enfermagem, Pesquisa em Enfermagem, Promoção da Pesquisa

## Abstract

The objective of the study was to identify the main challenges in conducting research with immigrants and refugees and to provide seven methodological and pragmatic strategies. The analyses presented, based on the Theory of Culture Care Diversity and Universality, are extracted from insights of the authors’ experiences as researchers and the literature. The main challenges are related to cultural, moral, political, and educational differences between researcher and researched; identification of the universe and sampling; access to informants through the barrier of distrust; and communication and language difficulties. Strategies to make research more successful involve: developing a thorough research protocol; creatively recruiting participants; developing strategies to facilitate communication; having a sensitive look; offering a structure of reciprocity; increasing trust, and triangulating research. The main methodological and pragmatic issues in studies with immigrants and refugees were explored, providing valuable guidance for future projects. However, in different migration situations, researchers must be aware of the possibility of other challenges arising during the investigative process.

## RESUMO

O objetivo do estudo foi identificar os principais desafios na condução de pesquisas com imigrantes e refugiados e fornecer sete estratégias metodológicas e pragmáticas. As análises apresentadas, embasadas na Teoria da Diversidade e Universalidade do Cuidado Cultural, são extraídas de *insights* das experiências dos autores enquanto pesquisadores e da literatura. Os principais desafios relacionam-se a diferenças culturais, morais, políticas e educacionais entre pesquisador e pesquisado; identificação do universo e amostragem; acesso aos informantes pela barreira da desconfiança; e dificuldade de comunicação e idioma. As estratégias para tornar a pesquisa mais exitosa envolvem: desenvolver um minucioso protocolo de pesquisa; recrutar participantes de forma criativa; elaborar estratégias para facilitar a comunicação; ter olhar sensível; oferecer uma estrutura de reciprocidade; ampliar a confiança e triangular a pesquisa. Foram exploradas as principais questões metodológicas e pragmáticas nos estudos com imigrantes e refugiados, fornecendo orientações valiosas para projetos futuros. Entretanto, em diferentes situações de migração, os pesquisadores devem atentar-se para a possibilidade de surgirem outros desafios durante o processo investigativo.

## RESUMEN

El objetivo del estudio fue identificar los principales desafíos al realizar investigaciones con inmigrantes y refugiados y proporcionar siete estrategias metodológicas y pragmáticas. Los análisis presentados, basados en la Teoría de Diversidad y Universalidad de la Atención Cultural, se extraen de *insights* de las experiencias de los autores como investigadores y de la literatura. Los principales desafíos están relacionados con las diferencias culturales, morales, políticas y educativas entre investigador e investigado; identificación del universo y muestreo; acceso a informantes a través de la barrera de la desconfianza; y dificultades de comunicación y lenguaje. Las estrategias para lograr que la investigación sea más exitosa implican: desarrollar un protocolo de investigación exhaustivo; reclutar participantes de forma creativa; desarrollar estrategias para facilitar la comunicación; tener una mirada sensible; ofrecer una estructura de reciprocidad; aumentar la confianza y triangular la investigación. Se exploraron las principales cuestiones metodológicas y pragmáticas en los estudios con inmigrantes y refugiados, proporcionando una valiosa orientación para proyectos futuros. Sin embargo, en diferentes situaciones migratorias, los investigadores deben ser conscientes de la posibilidad de que surjan otros desafíos durante el proceso de investigación.

## INTRODUCTION

The process of immigration and refuge is a contemporary phenomenon of global scope and for it to be characterized, distance, motivation and rupture have to be highlighted. Immigration is understood as a voluntary process, in which people cross borders with the prospect of obtaining better economic, educational, social, and cultural opportunities. On the other hand, refuge (or forced immigration) is characterized as flight from the country of origin for fear of persecution, violence, conflicts, or different circumstances that disturb public order. From this perspective, refugees need international protection^(1)^.

In 2022, estimates showed that one in every 30 people in the world was an immigrant. This corresponds to 281 million people displaced from their countries^(2)^. According to the Office of the United Nations High Commissioner for Refugees, at the end of 2022, around 108.4 million people had a refugee status, the largest number recorded in the history of humanity^(1)^. Facts such as the war between Russia and Ukraine, civil wars in Israel, Afghanistan, Syria, and Ethiopia, climate change and the extensive economic and political crisis in Venezuela cooperate to drive the current number of people displaced from their countries of origin^(1,2)^.

There is no room for doubt that immigrants and refugees are a particularly vulnerable group as they experience several factors (past and current) that determine their health condition. Poverty, lack of employment, low educational levels, precarious housing conditions, food insecurity, among others, are the main obstacles in the process of integration into a new society^(3)^. Furthermore, they face linguistic, cultural, social, political, and economic barriers – which result in greater morbidity and mortality – making immigrants and refugees over-reliant on social assistance and health services^(4)^. However, when seeking health services, they are faced with weaknesses in assistance that do not take the specific needs for culturally sensitive care into account^(5,6)^.

To overcome this gap, nurses and other professionals can base their practice on the Theory of Culture Care Diversity and Universality (TCCDU), proposed by Madeleine Leininger^(7)^. This is because this theory presents great concern with care based on respect for each person’s beliefs, values, and attitudes, within a cultural context. Moreover, it should be noted that the TCCDU combines theory and research method, distinguishing itself into different levels of abstraction and analysis^(7)^. Therefore, it can also be used within the scope of nursing research. In this regard, its aim is equally to support the investigative process, assisting in the development of a body of scientific and humanized knowledge capable of promoting culturally based, conceptualized, planned and operationalized care practices^(7)^.

It is clear that the theoretical and methodological approach to TCCDU assists in generating basic and applied knowledge in nursing science, given its potential for conceptual abstraction and its proximity to the cultural context in which people live and phenomena occur. Therefore, it was chosen to support this theoretical study, because its assumptions allow anchoring the proposition of strategies for the adequate development of research carried out by nurses, strongly considering the influence that aspects of the beliefs and culture of immigrants and refugees have on the data production and analysis.

Given the complexity of the phenomenon of migration and its consequences on the political, cultural and economic aspects of contemporary societies, this theme appears as recurrent in the scientific-academic context^(3)^. A major focus of current nursing literature addresses health-illness issues of immigrants and refugees, including quality of life^(8)^, mental health^(9,10)^, pregnancy and birth^(8)^, and health care within the scope of Primary Care^(11)^, of emergencies^(12)^, and hospitals^(9)^. Such studies have adopted different theoretical-methodological approaches, such as exploratory^(8)^, cross-sectional^(13)^, and descriptive^(9,10)^ designs, and qualitative^(3,9,12)^, quantitative^(10–13)^, and mixed methods^(8,11)^ approaches. Nevertheless, for decades there have been a series of challenges (especially for novice researchers) associated with research on the topic of immigrant and refugee’s health^(14)^, without, however, making an in-depth discussion about the methodological and pragmatic perspectives regarding the best strategies to promote research and enhance the current nursing scientific production in the field of migration studies.

In this sense, the question that arises is: what are the main challenges that nurse researchers encounter when developing studies with the immigrant and refugee population? What strategies can be used to enhance successful and valid research with this audience? Aiming at answering these questions, the development of this study was proposed to identify the main challenges in conducting research with immigrants and refugees and to provide seven methodological and pragmatic strategies.

## METHOD

The analyses presented here are based on critical thinking, insights and the authors’ experiences in nursing research with immigrant and refugee populations, as well as anchored in TCCDU^(7)^ and in the scientific literature on the themes of migration, health, and nursing research. The relevant literature was identified through searches of the Pubmed/MEDLINE, SCOPUS (Elsevier), CINAHL (EBSCOhost), and Web of Science (Clarivate Analytics) databases. The following keywords were used: “Emigrants and Immigrants”; “Refugees”; “Nursing”; “Nursing Research”; “Research Promotion”. The Boolean operators AND and OR were used to structure the searches.

The research took place in June 2023 and was limited to articles in Portuguese, English, and Spanish, given the researchers’ fluency in these languages. Texts published between 2010 and 2022 that focused on describing data collection and analysis processes in research with immigrants and/or refugees in the health context were considered. A manual search was carried out to expand the scope of selected studies. This allowed the addition of relevant articles that contributed to the results of this theoretical discussion and that had not been identified in the previously search on the databases.

After identifying the relevant texts, an attentive reading was carried out to seek information about the main challenges and the different strategies used by researchers in developing the studies. The information was summarized by three of the authors of this reflection who later came together to combine and compare the textual synthesis process. Throughout the whole process of text selection, reading, and analysis, the researchers prepared theoretical-reflective notes that recorded the main decisions and analytical interpretations. In case of disagreements or doubts about the challenges and strategies in research with immigrants, the most experienced members of the group were consulted for consensus. During this process, an audit trail was maintained, recording in theoretical and reflective notes the statements and propositions that emerged and were debated among the authors.

The reflective text is structured around two thematic axes, namely: “challenges inherent to nursing research with immigrants and refugees” and “strategies to enhance nursing research with immigrants and refugees”. In this section, methodological and pragmatic issues will be presented, that is, practical and realistic considerations that can enable nursing researchers to develop their research with this population group methodologically in a more successful manner.

As it was a secondary study that did not involve data collection with human beings, analysis by the Ethics Committee was not necessary, but procedures were considered to ensure that the primary studies used to construct the reflection had respected ethical principles.

## DEVELOPMENT

**Figure 1:**
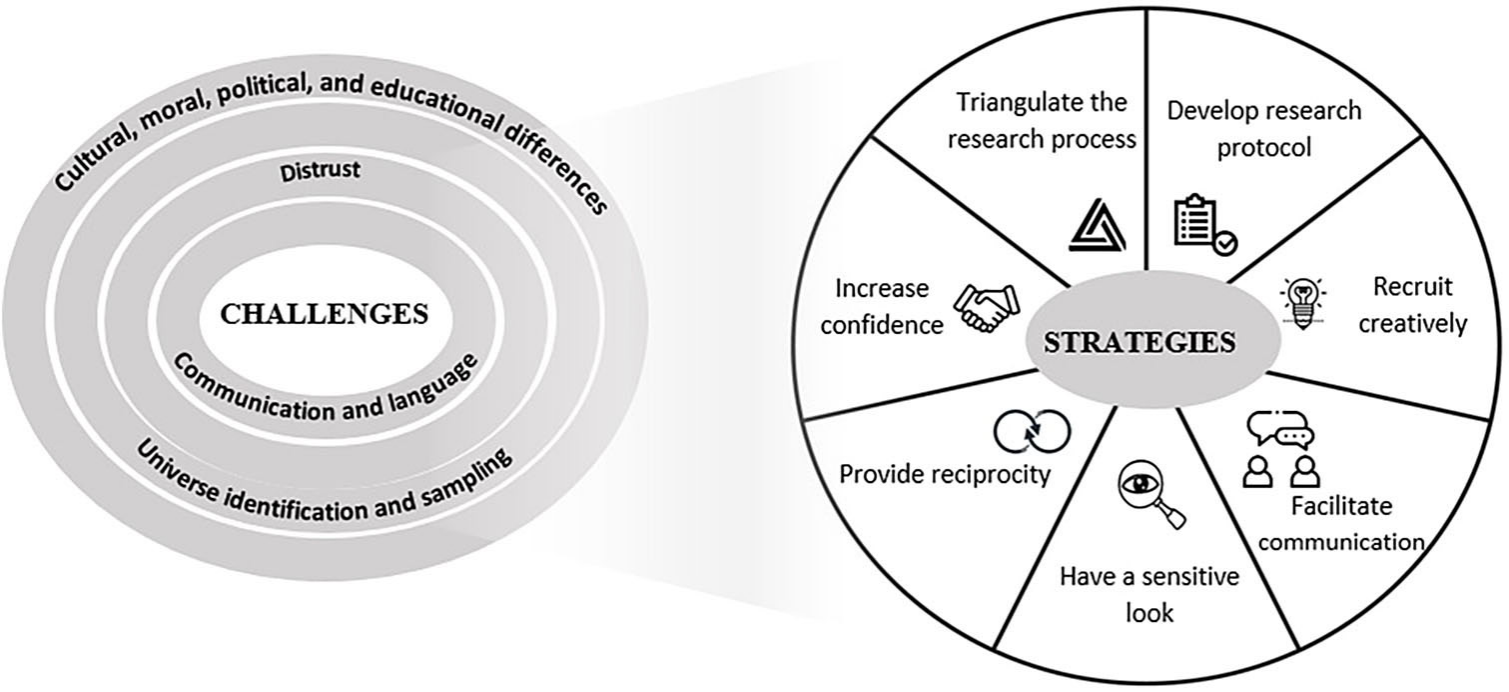
Challenges and strategies for nursing research with immigrants and refugees, 2023.

Nursing and health research for minority populations, such as immigrants and refugees, is a latent requirement of science, globally, in current times. Based on the authors’ experience as scholars in the area, the current literature on the topic and the TCCDU it was possible to identify the main challenges that researchers experience in practice when developing studies with immigrants and refugees, which involve, from the broadest to the most specific (from the outside to the inside in Figure 1), first the cultural, moral, political, and educational ones between researcher and researched; then, the difficulties in identifying the universe and sampling; next, the difficulties in accessing informants due to the barrier of distrust; and, finally, when accessing this population, the difficulty with communication and language.

Based on these challenges, pragmatic strategies (that is, practical and feasible) were identified and designed to enhance research, namely: developing a detailed research protocol; creatively recruiting participants; adopting strategies to facilitate communication; having a sensitive look; offering a structure of reciprocity; increasing confidence; and triangulating the research process. The representation of strategies in a circle indicates that there is no hierarchy within them, in fact they interconnect and complement each other (Figure 1).

### 1) Challenges Inherent to Nursing Research with Immigrants and Refugees

#### Cultural, Moral, Political, and Educational Differences

Cultural shock, resulting from moral, political, cultural, and educational differences between different countries, is an important aspect to be considered as challenging when establishing the relationship between researcher and researched. TCCDU points out that cultural diversity consists of the variety of standards, values, and symbols between different groups, societies and cultures^(7)^, with social structure strongly influencing diversity. Social structure is a dynamic process, of interdependent relationships, of different structural or organizational elements of society (i.e. religious, military, political, educational, technological, cultural, environmental and linguistic systems) that guide relationships^(7)^.

In some situations, immigrants and refugees may feel afraid that, when they tell the researcher about intimate aspects of their life and health, they will be judged, segregated, or exposed to situations of prejudice and xenophobia. This represents what in certain cultures is understood as the “public self” and the “private self”^(15)^. Therefore, interviewees tend to behave and dialogue as they believe that they can be socially, morally, politically and culturally better understood and not judged by native researchers. Additionally, if the content or style of an interview is culturally inappropriate, it will be misunderstood and perhaps terminated earlier^(14)^. It is important to consider that these situations are reflected in the accuracy of the data collected and in the way the information will be interpreted by the researcher^(14,15)^.

#### Universe Identification and Sampling

Research aims to discover general concepts and predict future behavior in a universe or population sample^(16)^. Therefore, to achieve success in investigations, whether epidemiological or interpretative, the first step is to adequately select a random or theoretical sample that is representative^(16)^. In the case of immigrants and refugees, the environmental context, that is, the geographic space/territory in which they are dispersed, is an interdependent component of the social structure^(7)^, and consequently, influences the identification of the universe and sampling for the development of studies.

It is necessary to consider that in research with immigrants and refugees there are several difficulties in selecting participants. Many of them do not live in identifiable ethnic communities, that is, they reside dispersed throughout cities and neighborhoods within the same city. There are few social assistance or health services that can compile the address and telephone information of immigrants and refugees. Furthermore, as they constitute a vulnerable group, there are important ethical considerations that make it difficult for researchers to access personal information. Another important point is that those who do not yet have documents will not be identified in formal records, compromising the possibility of accessing the address and telephone number. Therefore, numerical data on immigrants and refugees vary greatly between those considered “official” and those that are “real” and accessible to researchers^(14)^. Furthermore, follow-up studies (e.g. cohort or experimental) find the high turnover of addresses along the migration route as a barrier^(17)^.

#### Access to Informants: Barrier of Distrust

Aspects related to cultural diversity and the way people establish their relationships modulate the degree of trust they place in others^(7)^. Immigrant and refugee populations may be reluctant to participate in studies due to a lack of familiarity with research or because of previous experiences in which they have been exploited or deceived for research purposes. There may also be concerns or fears that confidentiality may be breached and that information provided for a study will be passed on to government officials or immigration authorities, particularly in refuge or undocumented situations. In addition, qualitative interviews are generally recorded and later transcribed in full for data analysis. However, this common procedure may not work with a population unfamiliar with the methodological tools of scientific research, understanding the recording as potentially threatening. Another challenge is related to intervention studies which, by their nature, require longitudinal engagement of participants, so that lack of trust constitutes a preponderant element for non-adherence in the long term and, therefore, a limitation^(11,16)^.

## COMMUNICATION AND LANGUAGE

TCCDU states that effective communication is essential for building positive relationships with patients during care and is a key element for adaptive changes to occur^(7)^. This has to be extended to the researcher-researched relationship. Communication and language barriers are fundamental aspects involved in the production of data in research of different methodological natures. In this case, it is not just a matter of mastering different languages. Communication goes further, involving habits, mannerisms, speed, and intonation, present in non-verbal and paraverbal communication. The literature suggests that immigrants and refugees prefer to be interviewed in their native language^(14,16)^ and that they also choose to use clear language without scientific jargon in the health area. In this regard, the interview and its style need to be close to the communication style of the group of immigrants and refugees under study, otherwise the answers may be circular or indirect, making data collection difficult^(14)^.

### 2) Pragmatic Strategies to Enhance Nursing Research with Immigrants and Refugees

#### Develop a Thorough Research Protocol

Developing thorough research protocols is important to minimize possible errors or unplanned situations that could impact the quality or methodological rigor of the study. When preparing research protocols for immigrants and refugees, investigators need to consider cultural uniqueness^(7)^ of the target group. To increase the reliability of the protocol, it can be validated through a pilot study or through the analysis of a panel of experts in multicultural research and/or in the methodological approach adopted. An alternative way to reduce the cost of a pilot study – but at the same time maximize the effectiveness of a panel of experts – is to bring immigrants and refugees who are community leaders to participate in the panel discussion. Being members of the community and knowing potential participants, these individuals can speak from the perspectives of the construct of culture and social structure and thus anticipate possible operational or methodological issues^(16)^.

#### Recruit Participants Creatively

The importance of employing creative and innovative recruitment and selection methods is highlighted, especially using members of the target community to be reached. These key people, often community leaders, can support recruitment. Furthermore, when the study population is quite heterogeneous in terms of ethnicity or age, for example, it is important to associate a variety of recruitment methods (pamphlets, face-to-face, media, social media) and different search locations (churches, health services, community organizations). This is relevant not only to increase participation, but also to ensure data variability and quality^(11,15)^.

Another strategy that has been used in studies with immigrants and refugees is the use of the technique called snowball. According to this technique, people with certain common characteristics are connected to a social network, made up of ties, and are therefore more easily identified by another member of the cultural community than by researchers^(16)^. This technique, despite being non-probabilistic, has achieved good results in reaching samples for quantitative^(11)^ and qualitative studies with immigrants^(3,15)^.

#### Triangulate the Research Process

Studies on migratory processes are sometimes still confined to imprecise and incomplete methodological approaches, as they are generally unable to cover cultural diversity^(7)^ and migratory mobility as a whole. To overcome this gap, triangulating the research process can be an important strategy. Triangulation involves the use of different research methods or different philosophical-paradigmatic approaches to study a phenomenon^(16)^. For example, researchers can employ a mixed methods approach to studies with immigrants and refugees, expanding the variability of the information collected, which is reflected in more accurate and complete data.

The data collection source can also be triangulated using data from interviews, questionnaires, field diaries, medical records, and databases^(14)^. In the experience of the authors of this reflection, during a multicenter study with immigrant and refugee families living in Brazil, Spain, and Portugal, combining the application of printed and online questionnaires allowed the expansion of data collection and the scope of the initially planned sampling.

In addition, to capture the breadth and complexity of the migration phenomenon, studies need to be longitudinal or involve immigrants and refugees at different stages of the migration process, as migration is a procedural transition, which includes pre-immigration, arrival, settling, integration, and acculturation^(14)^. That said, triangulating data collection with immigrants and refugees from different stages of migration or carrying out the research longitudinally, following them for a while, helps to understand the phenomenon in a more universal, comprehensive and plural way.

Finally, it is possible to triangulate the study informants, considering immigrants and refugees, their families, health professionals, educators, employers, community leaders and all those who can, in some way, increase the understanding of the phenomenon being investigated, analyzing it from different spectrums. Although in the area of nursing studies mostly involve themes inherent to the profession and the health-disease process, multidisciplinary teams of researchers, including anthropologists, sociologists, geographers, historians, epidemiologists, among others, can bring different perspectives to the study and the process of research triangulation.

#### Facilitate Communication

As already mentioned, the TCCDU states that an effective communication process is essential for building relationships^(7)^. In this sense, a strategy for allowing research with immigrants and refugees to be more successful involves considering linguistic diversity and, therefore, the integration of multilingual instruments for data collection. If the immigrant population does not yet master the language of the destination country, the use of instruments in different languages, the use of interpreters or, preferably, conducting interviews in the participant’s native language should be considered in the study design. This will facilitate interpersonal communication, the recognition of immigrants/refugees’ sense of identity, and the respect for their worldview^(7,15)^.

Regarding instruments, researchers should not assume that there are reliable and valid translations of standardized research instruments and measures. In fact, it is more common for translations to have been poorly done and not tested to ensure the validity of the use of dialects and linguistic expressions. Therefore, it is suggested that if interviews cannot be carried out in the participant’s native language, the use of interpreters is preferably adopted. However, the qualifications, timing, employment, duties, and training of interpreters used in the study must be planned well in advance^(18)^.

Employing the use of cultural mediators would be another important strategy to facilitate the communication process during data collection^(11,15)^. Such mediators are bicultural and bilingual research team members and, therefore, culturally competent. They are able to understand not only the worldviews^(7)^ of the participant group, but also the personal biases that researchers bring when interpreting different cultures. This way, the presence of mediators is relevant to resolve possible tensions felt between immigrants under investigation and researchers, which has the potential to promote a situation of exchange, sharing, and means of interpretation between subjects^(6)^.

Furthermore, it is important to encourage answers in qualitative research to be given especially in the form of everyday examples or through “storytelling”, also taking advantage of every opportunity to understand the phenomenon under investigation, including in moments when researcher and interviewee are introducing themselves or saying goodbye at the end of the interview^(14)^.

#### Have a Sensitive Look to the Participants

A sensitive look at the light of TCCDU is the same as saying a “culturally congruent look”. In this respect, it is necessary to consider that the family, and not just the individual migrant in isolation, has been the focus of some studies^(3,19)^ and, in these cases, the view needs to be expanded beyond the individual cultural aspect. It is necessary to consider, for example, that for Haitians, especially among refugees, there is the concept of “community families”, which are not restricted to spouses and their children. In fact, the family comes to be understood as people who live under the same roof^(19)^. These people have historical connections, as they experienced the same pain and difficulties, and when they find themselves in another country, they come together and care for each other, because above all they have the same ideals of maintaining and protecting family and life. For them, the concept of family is much more than blood connections – the emotional bonds built constitute the foundation for facing the difficult conditions experienced in everyday life. In the meantime, researchers need to look sensitively at the new social configurations and structures that form and strengthen in the lives of immigrants and refugees.

Therefore, one cannot genuinely understand the migratory process and the migrant without knowing and questioning the social, political, economic and scientific status given by the State or academia to this population group. It is important that the nurse researcher, as a sensitized “intellectual craftsman”, leaves his/her own precepts in suspension and adjusts his/her research questions, concepts, and data collection instruments to this object under investigation and its cultural, structural, educational, and family^(7)^ specificities, and the nurse’s sensitivity must move towards avoiding cultural generalizations, which reinforce stereotypes, increase prejudice, and hinder the development of a sensitive and unique perspective for each person^(7)^.

Something that can help nurses develop this more sensitive and even comprehensive view of migrant populations is the adoption of powerful theoretical frameworks, which function as lenses to support the researcher in interpreting their findings. For example, the TCCDU by Madeleine Leininger, for decades, has been available in the literature to support nurses in the scope of practice, teaching and research with culturally different populations^(7)^.

However, it is important to highlight that, to train a nursing researcher, the person goes through the professional training process at the university, at which point they must receive concepts that equip them for culturally competent care^(20)^. TCCDU emphasizes the importance of preparing clinical nurses and educators to meet the needs of several populations. This approach aims to prevent practices of cultural imposition, ethnocentrism, and cultural conflicts in clinical environments^(21)^, which is reflected in the researcher’s attitude when working with immigrant and refugee populations.

#### Provide a Structure of Reciprocity

Another useful strategy for gaining access to immigrant and refugee communities is to provide health-related services. This has the potential to increase visibility and improve the researcher’s reputation within the group, considering that immigrants and refugees generally have an effective relationship network among them^(14)^. For example, before starting data collection, nurse researchers can organize a small health fair, with blood pressure measurement, blood glucose measurement, and provision of health guidance during the meeting of a non-governmental organization that serves the target population, or even in front of a church that brings together a significant number of immigrants and refugees.

Furthermore, researchers must make themselves available to interviewees to assist them with small demands, such as, for example, information about the national health system, scheduling of appointments, cultural tips about the city, information about job offers, vacancies for courses in universities, or technical courses. This reciprocity constitutes an ethical commitment of the researcher and is relevant given the context of vulnerability that immigrants and refugees tend to experience in the host country, as well as being a response to the trust placed in the researcher.

All of these ways of offering reciprocity need to be in harmony with people’s cultural beliefs, practices, and values^(7)^. In this context, researchers must be careful to consider the differences and similarities between different cultures when offering health services and care to the immigrant and refugee population to be researched.

#### Increase Confidence

TCCDU highlights the importance of building positive relationships between professionals and patients^(7)^. Here, it is reiterated that such construction must also occur between researcher and researched. To that end, using relationships with community-based organizations, such as health services and/or non-governmental organizations, besides including members of the target community of the immigrant and refugee group, are essential to building trust and recruiting participants^(11)^. Individual interviews and/or repeated meetings may also be necessary so that the participant has sufficient trust in the researcher, which will allow more robust, precise and reliable data to be obtained^(14)^.

The order of questioning is also important. It is more effective to ask the “least threatening questions” first^(14)^, for example, starting with questions related to people’s general health tends to result in better answers than questions about sociodemographic data. Furthermore, it is suggested that researchers negotiate with ethics committees so that there is permission to obtain verbal consent at the beginning of the interview, with signatures at the end. This can not only reduce the difficulties researchers face in obtaining informed consent, but also increase the level of trust and comfort among immigrants/refugees in expressing their agreement to participate in research^(11,16)^.

Another noteworthy point refers to the fact that in studies involving immigrants and refugees, the return of data to the researched populations, institutions and social movements involved is an ethical commitment of the researcher and implies “sharing the benefits found” useful to improve people’s lives^(16)^. This citizen science movement, as long as it is carried out in language accessible to the population, also has the potential to increase the confidence of immigrants and refugees in scientific research and researchers, promoting a more receptive culture to scientific investigation among them.

## FINAL CONSIDERATIONS

This study presents the challenges and strategies for developing research with immigrants and refugees. The main challenges are related to cultural, moral, political, and educational differences between researcher and researched and access difficulties to informants due to the barrier of distrust and those inherent in communication and language. Seven pragmatic strategies were designed to enhance and make research more successful. The analyses carried out can support undergraduate and postgraduate students, members of research groups and teachers in operationalizing reflections and discussions regarding the importance of planning and developing research concerned not only with its quality and methodological rigor, but which value this population’s specificities and needs.

Finally, the reflections made here on methodological and pragmatic issues provide nurses with valuable guidance for future projects. However, this analysis has limitations. The guidance is not comprehensive enough to cover all potential challenges that researchers may encounter in the field, nor is it appropriate for all populations or immigration situations. Furthermore, the fact that research with immigrants and refugees is still incipient in the area of nursing led to the limitation of bringing the theoretical-reflective discussion closer to the practice of research carried out by nurses. Therefore, more nursing studies are necessary to expand knowledge about the difficulties experienced in practice and thus fill the gaps and resolve uncertainties that still remain. The researchers must keep in mind the uniqueness of the immigrant and refugee populations with which they are working and be flexible to adjust their research projects as they are carried out, without violating ethical principles and scientific rigor.
